# Phytohormones unlocking their potential role in tolerance of vegetable crops under drought and salinity stresses

**DOI:** 10.3389/fpls.2023.1121780

**Published:** 2023-02-28

**Authors:** Jun Chen, Xin Pang

**Affiliations:** Faculty of Horticulture Science & Technology, Suzhou Polytechnic Institute of Agriculture, Suzhou, China

**Keywords:** metabolic mechanisms, irrigation practices, sustainable yield, water needs, crops

## Abstract

Globally, abiotic stresses are drastically reducing the productivity of vegetable crops. Among abiotic stresses, drought and salinity are more challenging constraints for the sustainable production of vegetables. A great variety of vegetables are facing dry and hot summer spells, poor water availability, and higher salinity mainly due to irrigation with brackish water. Vegetables are considered higher water-dependent crops, requiring water for proper growth and yield. Drought and salinity impair plant metabolism. The disruption in plant metabolism leads to a reduction in growth, developmental processes, and ultimately crop yield. Appropriate management measures are needed to cope with the adverse effects of drought and salinity. Different agronomic and molecular approaches contributed to improving tolerance. Therefore, the present review significantly explores the impact of phytohormones on vegetable crops under drought and salinity stresses. Phytohormones (salicylic acid, melatonin, jasmonates, Brassinosteroids, ascorbic acid, and numerous others) can be sprayed for improvement of plant growth, yield, and photosynthetic pigments by modulation of physiological and biochemical processes. In this manner, these phytohormones should be explored for sustainable production of vegetable crops growing under abiotic stress conditions.

## Introduction

Phytohormones are considered plant-protecting hormones under abiotic stress conditions. Different phytohormones are well-known management strategies, which act as stress-relieving bioactive compounds in vegetable crops (Fahad et al., 2015; Altaf et al., 2022a). The exogenous spray of numerous phytohormones can reduce the drought and salinity stresses and also improve the plant defense mechanism focusing on sustainable production. Fascinatingly, phytohormones are more effective for the reduction of challenges that occur from stressful conditions at any growth or developmental phases even from germination to plant senescence. These hormones are contributing to numerous signaling and transduction pathways through hormonal reception and regulatory actions (Hu et al., 2012). The membrane receptors, ionic networks, reactive oxygen species (ROS) indications, and mitogen-activated protein kinase (MAPK) indications are noticeable to numerous fundamental utilities in the synchrony by phytohormones to cope with the negative effects of abiotic stress. Understanding the interactive mechanism of phytohormones and transcriptomics can be effective for the development of tolerant germplasm of vegetables (Diao et al., 2015; Mangal et al., 2022). Modulation of physiological and photosynthetic pigments was found to be helpful for an increase in plant yield growing under water stress and saline conditions. The susceptible germplasm can also become higher yielding by sufficient use of phytohormones based on genetic makeup and climatic conditions of the characterized germplasm, as reported by Forni et al. (2017). However, the impact of phytohormones on the regulation of secondary metabolites and other signaling molecules needs further investigation for better understanding. For the development of tolerant germplasm, traditional breeding ways are time-consuming and not specific. However, the application of phytohormones is more effective for the alleviation of abiotic stress tolerance.

Plants growing under field conditions could be exposed to multiple stresses, which can damage the crops’ yield (Glick, 2012). Severe climatic conditions in summer, irregular nutrition management, and unavailability of irrigation water are causing stunted growth and poor crop yield. Sustainable agricultural crop production is drastically affected by numerous biotic stresses (such as insects, pests, and disease) and abiotic stresses (like drought, salinity, temperature extremes, humidity, light, ultraviolet radiations, mineral nutrition deficiencies, and heavy metals) (Akram et al., 2017; Shakoor et al., 2017). Drought and salinity are considered more destructive conditions, extensively affecting growth, developmental stages, and yield. Plants can change their defense system against stressful conditions to regulate metabolism, growth, and development (Ahmad et al., 2008). Vegetable crops are potentially growing under diverse environmental conditions by natural acclimation, as well as numerous adaptation strategies. However, these approaches may not be sufficient to reduce losses from variations in climate change (Shahid et al., 2021; Zhang et al., 2022a). The severity of abiotic stress is mainly based on the type of species and intensity and duration of stress (Zhang et al., 2022b). Stressful conditions cause variations in plant physiological and biochemical processes, either reversible or irreversible. However, these constraints affect vegetable crops primarily, which are susceptible to abiotic stress (Parveen et al., 2020). Presently, vegetable crop demand is higher; therefore, it is necessary to develop some excellent approaches or tolerant germplasm to tackle the severity of drought and salinity stresses. Drought and salinity stresses are critical global concerns and harm the sustainable production of crops. Irrigation water resources are depleting due to climate change, urbanization, and industrialization (Gruda et al., 2019). Soil salinity is also increasing, mainly due to irrigation with poor-quality and brackish water. The unavailability of quality water in various regions is causing salt accumulation in the soil, which further translocates toward the root zone of vegetable crops. It has been estimated that approximately 20% of global land is negatively affected by salt extremes (Forni et al., 2017).

Vegetables are considered an essential source of the human diet because they are rich in dietary fibers, vitamins, antioxidants, and minerals. Their consumption is also due to good taste, excellent texture, and religious value (Gamalero and Glick, 2022). Global vegetable production in 2020 increased by nearly 66%, from 447 to 1,130 Mt (FAO, 2021). Farmers are investing considerable efforts in improving vegetable production and nutritional aspects under stressful environments (Gruda et al., 2019). The severity of drought and salinity is mainly based on different climatic constraints like the distribution of solar radiation, the need for evapotranspiration, and the retention of soil moisture content (Sabir et al., 2022). Hence, numerous agricultural practices and breeding approaches can be employed for the alleviation of tolerance in vegetable crops against drought and salinity.

Plant researchers urge sustainable management practices to increase vegetable production under drought and salinity stresses (Ahmad et al., 2010; Checker et al., 2018). The exogenous application of phytohormones is a more promising approach to cope with the adverse effects of drought and salinity for sustainable vegetable production. The involvement of phytohormones is attracting much attention from plant researchers due to their multifunctioning behavior against drought and salinity stresses. However, their utilization is still limited in vegetable crops growing under drought and salinity. Therefore, the present study elaborates on the utilization of phytohormones in vegetable crops under drought and salinity stresses. Deep insights into physiological, biochemical, and molecular basis were also explored in the vegetables to cope with the adverse effects of drought and salinity.

## Phytohormones are major modulators of plant responses to drought and salinity

Vegetable production is low in different growing areas due to water deficit and salinity. Restricted growth and low yield are due to the unavailability and shortage of water and excessive salt accumulation in the root zone of plants. The higher uptake of Na^+^ through roots by xylem vessels resulted in restriction in the uptake of nutrients and minerals necessary for sufficient growth and yield (Maksimovic and Ilin, 2012). Salinity, sodicity, and water stress revealed adverse effects on the growth, yield, and quality of vegetable crops. Higher accumulation of salts disturbed the soil structure, texture, porosity, and permeability of water, which ultimately reduces the productivity of vegetable crops (Malhi et al., 2021). Soil provides better anchor and acts as a reservoir of mineral nutrients necessary for better growth, development, and yield. Therefore, the development of mechanistic approaches is needed to minimize the damaging effects of drought and salinity in vegetable crops. Drought and salinity affect vegetable crops, causing restriction in growth with poor yield (Hossain et al., 2022). Drought stress and excessive Na^+^ accumulation are causing a disturbance in the metabolism of vegetable crops. Moreover, the osmotic potential of plants is also adversely affected due to drought and excessive salt accumulation in different plant cells and compartments (Zaidi et al., 2015). Alterations in metabolism and disturbances in osmotic potential are the leading causes of restricted growth and low yield and sometimes complete or partial death of a plant (Neha et al., 2021).

In addition to supporting signaling pathways, endogenous plant hormones are critical in the response to drought and salinity. Phytohormones play a major role in mediating how plants respond to osmotic adjustment under stress conditions. Small signaling molecules called phytohormones have a significant impact on almost every aspect of the development of plants. The methods of action taken by different hormones for various activities may be very different. Furthermore, it is well recognized that even a single hormone can have an impact on a wide range of cellular and developmental processes or that multiple hormones can regulate a single function concurrently. Phytohormones protect and control plants from biotic and abiotic stresses. As a result, phytohormone application aims to expand crop stress research in the future (Ahmad et al., 2008).

## General signs and ion toxicity under drought and salinity stresses

**Figure 1:**
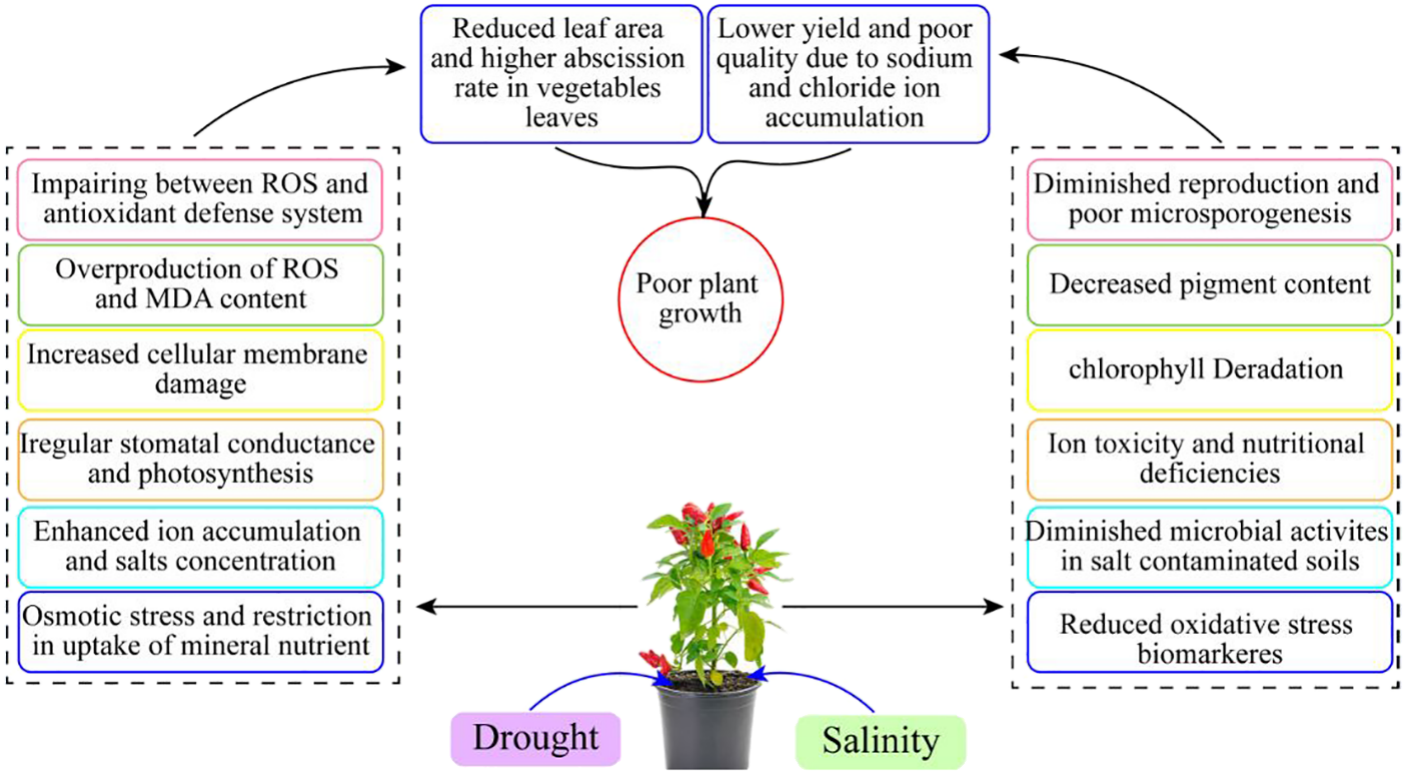
Adverse effects of salinity in vegetable crops.

Drought and salinity stress decrease the uptake of Ca^2+^ and K^+^ in vegetable crops, which is the primary reason why nutritional imbalances occur in plants. Plant physiology and morphology are also affected by numerous stresses and thus are susceptible to drought and salinity stresses (Rodrıguez et al., 2005). The initial response of vegetable plants under drought and salinity is the dropping of leaves or the initiation of leaf senescence. After that, a reduction in fresh and dry weights may also be considered an early response of plants growing under water shortage and salinity stress conditions (Zhu, 2002). The decline in fresh and dry weights ultimately reduces the plant yield. Yield reduction is evident in vegetable crops growing under drought and salinity stresses (Alian et al., 2000). However, a reduction in yield can also be a responsive mechanism, especially in aerial plant parts (Sharma et al., 2011) (**Figure 1**).

Plants have been categorized into two main groups, halophytes and glycophytes. It has been reported that halophytes are more tolerant than glycophytes (Zhang et al., 2017). The potential of halophytes was much imperative, and higher survival and reproduction rates were observed as compared to glycophytes due to improved root architecture, regulation in stomatal conductance, balanced nutrition, improved metabolism, and distinctive genetic makeup (Gao et al., 2018). Halophytes can tolerate approximately 200 mM of NaCl because, at this level, glycophytes cannot survive. Furthermore, the halophyte group constitutes a 1% proportion of global flora, and the individuals of this group were grown naturally (Patane et al., 2013). Leaf growth, especially leaf area, is also considered an initial response in stressful conditions within plant cells and compartments. Numerous other signs include leaf scorching from tip and margins, yellowing and bronzing, leaf dropping of leaves, dieback in twigs, necrosis, blackening, and burning (Bernstein et al., 2004).

Different ion movements continue within plant organelles and compartments under normal conditions. Higher regulation of cytosolic K^+^ and Na^+^ ratio was recorded in the vegetable crops grown under favorable conditions (Zhu and Gong, 2014). Under salinity and drought conditions, ion balances are disturbed, and abnormal movements of ions continue until the availability of favorable conditions. Water-deficit conditions increased the accumulation of salts in the root zone (Sattar et al., 2021). Excessive Na^+^ in the root zone and its translocation to other plant parts are also improved. Na^+^ and K^+^ channels are also present in the xylem vessels. The discrimination of both ions is necessary, although both are similar in power to hydrated ions, and their discrimination is difficult for plants. However, some transporters of ions with high-affinity potassium transporters (HKTs) are more effective for the discrimination and movement of ions through xylem vessels in all plant parts. Furthermore, some proteins, such as integrated membrane proteins, are also involved in the regulation of solute movements within plant cells and compartments (Ahmad et al., 2008). Moreover, these transporters and proteins are specific for ion regulation; for example, some are specific for the discrimination of Na^+^ and others for the discrimination of K^+^. Hence, it has been reported that regulation of Na^+^ and K^+^ is necessary for sufficient plant growth, development, and yield of potatoes (Kamran et al., 2021).

Recently, vegetable crops are facing numerous biotic and abiotic stresses; however, a single abiotic stress is also sufficient for the drastic reduction in crop yield. The water shortage and excessive salt concentrations show a direct effect on the reduction in vegetable crop yield (Lin et al., 2006). Any plant parts, even underground or aerial parts, can be damaged due to low soil moisture levels and excessive salts (Li et al., 2022). Under stressful conditions, vegetable plants and their response to drought and salinity are mainly based on the type of species, cultivars, and even landraces. It has been studied that Cl^−^ ions are effective for the catabolism of numerous enzymatic and non-enzymatic activities, and these are also known as co-factors for the regulation of the photosynthesis process (Rodríguez-Delfín et al., 2011). The behavior of sensitive and tolerant germplasm of vegetable crops toward Cl^−^ is more different. The excess of Cl^−^ is toxic; however, Na^+^ is more toxic than Cl^−^. Numerous genes are also involved in regulating Cl^−^ produced in plants. Aquaporin has also been involved in the characterization of numerous genes that contributed to the regulation of Cl^−^ efflux, which has significant involvement in the sustainable production of crops.

## Avoidance mechanism of vegetable crops against stressful conditions

**Figure 2:**
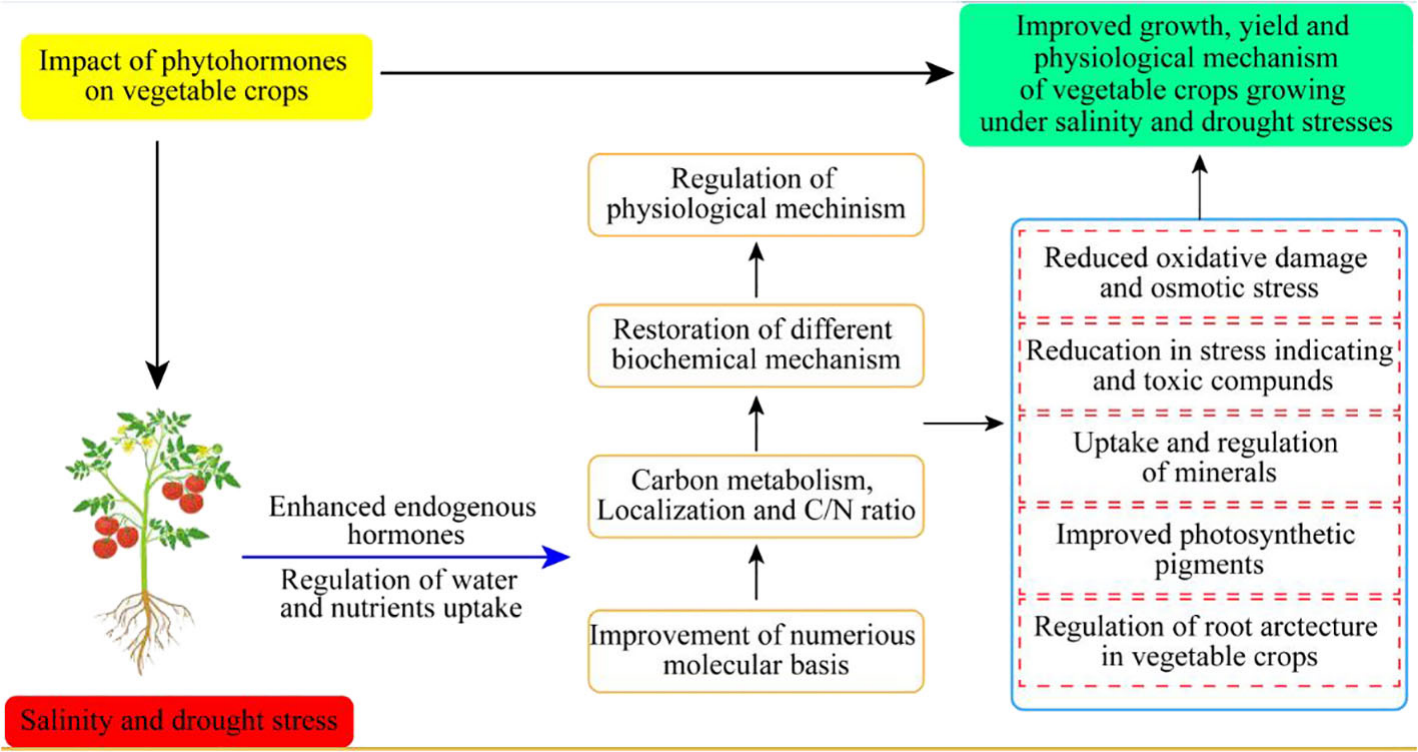
Critical stages of irrigation water at different growth stages of vegetable crops.

Salt exclusion and excretion restrict the salt’s access to the xylem vessels of vegetable crops. The exclusion of salts like Na^+^ and Cl^−^ *via* roots revealed that the storage of Na^+^ and Cl^−^ in leaves is not at a toxic level (Andre et al., 2009). However, their increased concentration disturbed physiological mechanisms, further resulting in leaf drop (Sobhanian et al., 2011). Grafting will be successful in numerous vegetable families and species like Solanaceae and Cucurbitaceae. Rootstock and scion combination contributed to the avoidance of salt mechanism in vegetable crops (Colla et al., 2010). Excessive salts in the root zone further translocated toward other plant parts. However, salt translocation can be reduced and not transported toward leaves. The rootstock’s basal portion can absorb the salts (Giordano et al., 2021). Therefore, it has been distinguished that the rootstock and scion combination is most necessary for alleviating salinity in vegetable crops (**Figure 2**).

Salt exclusion is a variety-specific character in vegetable crops, and the higher exclusion of salts is the capability of a specific variety. However, this mechanism does not reveal the tolerance mechanism in vegetable crops. The avoidance mechanism of drought and salinity is also based on the root architecture of vegetable crops. Zhang et al. (2019) reported that grafting improves plant performance under drought and salinity stress in tomatoes. Similarly, phytohormones could improve the Solanaceae vegetable crop performance.

## Phytohormones and gene expression under drought and salinity stresses

Expression of genes related to drought and salts is a more imperative utilization for the development of tolerant germplasm. Different genes and their expression in agronomic crops are widely discussed in the literature; however, in vegetable crops, this molecular phenomenon is still in progress. Most functional markers are related to numerous genes involved in the stress tolerance mechanism of vegetable crops (Ahmad et al., 2008). Gene expression potentially contributes to the development of salt-tolerant germplasm. Characterization of drought- and salt-tolerant and susceptible germplasm is a prerequisite for the sustainable production of vegetable crops (Malhi et al., 2021). Wild germplasm had more significant variation in genetic makeup and novel alleles, which can be explored to develop salt-tolerant germplasm. Numerous resistant genes can be identified, and further genome editing and transformation can be helpful for the development of tolerant germplasm in vegetable crops (Malhi et al., 2021). However, the expression of genes can be regulated by exogenous and endogenous improvements of phytohormones for the increase of tolerance against stress drought and salinity. Phytohormones are involved in the upregulation of transcriptomics of ATPase. Moreover, they were also involved in the reduction of the expression level of *PpATG* for the regulation of numerous morpho-physiological and biochemical activities in cucumbers. Moreover, similar findings were also reported by Parveen et al. (2021) for the expression of genes related to the tolerance mechanism.

## Management approaches for mitigation of drought and salinity in vegetables

Different management approaches comprised of proteomics, marker-assisted selection, genome characterization, genome editing, genome mapping, quantitative trait locus (QTL) mapping, genomic editing, and genomic transformation are promising molecular bases for salinity and drought tolerance in vegetable crops (Saidi and Hajibarat, 2020). Furthermore, the molecular bases can be utilized for the backcrossing of genes present in wild species toward offspring or landraces. The first genome map was developed in the 1980s on potatoes in relation to sexual recombination regularities. Plant breeders have successfully characterized disease-tolerant genes in potatoes (Byun et al., 2007). Moreover, numerous economic traits were detected in potatoes. Furthermore, “NL25” is one of the functional markers with excellent capability to identify candidate genes related to tolerance characteristics of potato warts (Saidi and Hajibarat, 2020).

## Phytohormones and vegetable crops under drought and salinity stresses

Phytohormones have the potential to enhance vegetables’ growth and development by interacting with numerous processes responsive to stressful conditions (Groppa and Benavides, 2008). Phytohormones have the capability to improve the defense system of vegetable crops growing under drought and salinity stresses. Plants activate their defense system against adverse climatic conditions for their survival (Choudhary et al., 2012). Therefore, supplementation of phytohormones boosts the immune system of plants growing under drought and salinity stresses. Plant defense system comprises the activation of enzymatic compounds (i.e., superoxide dismutase (SOD), peroxidase (POD), and catalases (CATs), non-enzymatic activities (i.e., ascorbic acid (AsA), phenolic content, and different sugars), osmolytes (i.e., glycine betaine (GB), ascorbate peroxidase (APX), and proline), and oxidative stress-indicating activities (ROS, malondialdehyde (MDA), and hydrogen peroxide (H_2_O_2_) (**Table 1**). Therefore, the impact of phytohormones on vegetable crops is imperative and needs more investigation on the molecular level to enhance plant tolerance (Diao et al., 2015).

**Table 1:** Role of different antioxidant activities in drought and salt tolerance mechanism of vegetable crops.

Bioactive molecules	Key findings	References
ROS, MDA, and H_2_O_2_	The activation of these activities is more toxic for plants. Chances of membrane injury increased under stress.	Sobhanian et al. (2011)
Electrolyte leakage	Membrane injury increased due to stress conditions because membrane damage enhanced due to the production of lipid peroxidation.	Zhang et al. (2013)
SOD, POD, and CAT	Toxic ROS, MDA, and H_2_O_2_ scavenging are made by CAT activity naturally. These are scavengers of toxic compounds, and their activation also improved the defense system of plants growing under stressful environments. These are helpful to disturb the O_2_ to form H_2_O_2_ and remove the harmfulness of superoxide anion.	Zhang et al. (2013)
APX and glutathione	Ascorbate activity enhanced the plants’ tolerance mechanism. These are effective to decrease the H_2_O_2_ production in vegetables against osmotic stress and oxidative injury. H_2_O_2_ and its derivatives are rapidly decreased by glutathione. These have better scavenging capability under stress conditions.	Wu et al. (2018)
Proline and GB	These osmolytes are considered signaling molecules against stress conditions. Proline and GB are known as antioxidant profiling that improves drought and salt tolerance in vegetables. Proline may act as a signaling molecule in order to maintain osmotic regulation. Oxidative injury is regulated by the production of proline and GB.	Zhang et al. (2013)

ROS, reactive oxygen species; MDA, malondialdehyde; SOD, superoxide dismutase; POD, peroxidase; CAT, catalase; APX, ascorbate peroxidase; GB, glycine betaine.

### Brassinosteroids

These are more emerging, eco-friendly, and multifunctional plant hormones involved in regulating physiological mechanisms occurring within the plants (Mumtaz et al., 2022). Plant researchers and physiologists are working on utilizing these plant hormones for sustainable crop production. Alhaithloul et al. (2020) reported that Brassinosteroids (BRs) are more effective for plants growing under drought and salinity stress environments. It has been studied that BRs enhanced seed germination, root growth, seedling development, cell expansion and differentiation, ripening of fruits, leaf senescence, and reproduction of floral parts of vegetable crops (Bhandari and Nailwal, 2020). Moreover, in the findings of Kaya (2021), it has been discovered that BRs can improve growth traits, mineral content, antioxidant activities, and osmolytes and protect from membrane injury. Similarly, Shahid et al. (2011) evaluated that BRs elevated pea productivity against drought and salinity. Thus, it has been confirmed that BRs effectively elevate salinity tolerance in vegetable crops. BRs are effective for amelioration of drought and salinity tolerance in numerous vegetable crops like tomatoes (Jangid and Dwivedi, 2017), cucumber (Jakubowska and Janicka, 2017), and radish (Ramakrishna and Rao, 2015). Furthermore, elevated enzymatic activities like SOD, POD, CAT, and improved metabolites were recorded with exogenous application of these phytohormones. Moreover, improved physiological systems and reduction in oxidative injury were also observed in numerous vegetables, i.e., tomatoes (Jordan et al., 2020), peppers, and cucumbers (Per et al., 2017; Fahad et al., 2019), by application of BRs. Abiotic stress tolerance can be mitigated in the radish by supplemental use of BRs. Reduction in the over-generation of ROS, MDA, H_2_O_2_, and electrolyte leakage indicated that BRs are stress-relieving compounds for radish plants growing under stressful conditions as studied by Ramakrishna and Rao (2015). Furthermore, the increase in plant defense indicated that BRS is effective for the improvement of the plant immune system against harsh environments. In another study by Jakubowska and Janicka (2017), it has been indicated that BRs are much more effective for abiotic stress tolerance, as a similar tolerance mechanism was reported in the cucumbers. The exogenous spray of 24-EBRs on cucumbers improved the gaseous exchange processes and all its related traits, chlorophyll fluorescence, starch, soluble sugars, and rubisco activities. Therefore, it is much more effective for higher-yielding vegetable crops growing under normal and abiotic stress conditions. Similarly, in the other research by Choudhary et al. (2012), it has been studied that free radicle-scavenging potential in radishes was improved with enhanced antioxidant potential along with improvements in morphological traits of roots under heavy metal (copper) excess. From previous literature, it has been indicated that BRs are the more effective, eco-friendly, naturally occurring substances that might be extensively utilized for the reduction of drought and salinity stresses in vegetables.

### Jasmonates

This group is comprised of methyl jasmonate (MeJA) and jasmonic acid (JA), which have been explored for their impacts on vegetable crops (Dar et al., 2015). Deprivation of photosynthetic pigments and tuber formation can be regulated under the exogenous application of JAs, as studied by Viswanath et al. (2020). The exogenous spray of this plant hormone improved sugar beet growth and defense system under drought (Ghaffari et al., 2019). Importantly, the exogenous JA application improved the endogenous production of JAs, and consequently, it can be used for hormonal regulation (Shahzad et al., 2015). MJ improved the drought resistance in cauliflower by improving oxidative bioactive compounds (Wu et al., 2012). Therefore, it has been exhibited that vegetable production can be increased with JA supplementation. JAs strengthen the defense system against environmental stresses in horticultural crops (Dar et al., 2015). These are significant for horticultural crops growing in areas with drought (Ge et al., 2010) and salinity (Pedranzani et al., 2003). Environmental threats can be regulated by the application of JAs. Similarly, Zou et al. (2017) revealed that the defense mechanism of plants was improved under environmental stresses like waterlogged conditions in peppers. JAs have good potential as a regulatory mechanism of vegetable crops against drought and salinity stresses. Abouelsaad and Renault (2018) reported that ROS mediation can be improved with JA because ROS is an indication of stress occurrence in tomato plants. Manan et al. (2016) reported that MeJA had the good capability to enhance the yield-related traits of tomato cultivars growing under elevated salinity as evaluated by Manan et al. (2016). The exogenous spray of MeJA on peas growing under stressful situations results in the improvement of indigenous hormonal levels of JA (Shahzad et al., 2015). Cauliflower grows under water-deficit conditions, facing challenges in growth at the seedling stage and poor yield at the reproductive stage. Wu et al. (2012) examined whether MeJA potentially triggered both oxidative and non-oxidative activities. Absorption and uptake of heavy metals were decreased in eggplant through the exogenous application of MeJA as supplementation (Yan et al., 2015). Seed priming is also an effective way to reduce challenges due to stress conditions. Therefore, it has been recorded that JA contributed to the increase in the germination of okra seeds, increase of seedlings, improved level of osmoprotectants, defense activities, photopigments, ROS reduction, lessening of H_2_O_2_, and low MDA level against salinity as studied by Iqbal et al. (2022). Exogenous application of MeJA on peppers improved osmolyte generation, oxidative and non-oxidative bioactive molecules, and metabolism and also improved the uptake of minerals *via* roots. Furthermore, decreases in MDA, H_2_O_2_, electrolyte leakage, and ROS were also reported by supplemental application of MeJA in the peppers. Therefore, it has been considered that JAs are suitable phytohormones for the mitigation of adverse effects of salinity and water-deficit conditions in horticultural crops.

### Salicylic acid

This is a phenolic-based hormone that contributes to the elevated growth and yield of vegetable crops grown under drought and salinity environments, mainly by improving the plant defense system (Khan et al., 2015). Similarly, in another vegetable crop (pea), different concentrations of salicylic acid were applied exogenously (nearly 1–4 mM) under salinity conditions (50, 100, and 150 mM of NaCl) (Saidi and Hajibarat, 2020). In this study, it has been noted that salicylic acid improved pea growth, yield, enzymatic and non-enzymatic activities, and osmolytes. Salicylic acid (SA) (300 ppm) improved the mineral content in garlic and decreased Na^+^ uptake and translocation to other plant parts. Therefore, it has been considered that SA is helpful for vegetable crops growing under drought and salinity stresses (Shama et al., 2016). Similarly, in another study, nearly 0.11 mM of SA improved the tolerance of potatoes against abiotic stress (chilling). Priming seeds with salicylic acid at 100 mg/L is an effective strategy for the mitigation of adverse effects of salinity in cucumber (Rehman et al., 2011). The use of salicylic acid is an effective strategy for tolerance of abiotic stresses in vegetable crops, i.e., potatoes (Li et al., 2019), bell pepper (Zhang et al., 2020), spinach (Gilani et al., 2020), and peppermint (Ahmad et al., 2018). Spraying 1 mM of SA on tomatoes growing under heat stress resulted in an improved process of gaseous exchange, good water use potential, enzymatic activity generation, non-oxidative activation, and reduced oxidative stress conditions as studied by Zulfiqar et al. (2021). Moreover, biomass reduction on a fresh or dry basis was decreased, ultimately reducing the yield because of salinity and drought stresses. Furthermore, disruption in photopigments, photosynthesis disturbances, and irregularities in the functions of stomata are causes of osmotic stress. Therefore, it has been explored that regularities in the process of photosynthesis and stomatal function are important by application of different levels of SA. Similarly, in other findings, nearly 0.1 mM of SA enhanced the fresh and dried biomass, regulated photosynthesis, generation of oxidative and non-oxidative compounds, regulation in electrolyte leakage, protection from membrane injury, efficient water use potential, and excellent anatomical responses (Galviz et al., 2021). Moreover, it has been recorded that SA had the capability to mitigate challenges that occur from drought and salinity stresses by reduction of oxidative and osmotic injuries (Kaya, 2021).

### Polyamines

The polyamine (PA) group from phytohormones primarily comprised spermidine, putrescine, and spermine having a lower molecular weight (Ahmad et al., 2012). Several physiological and biochemical processes were administered through polyamines by improving root, leaf differentiation, pollen viability, flower development, fruit growth, gene transcription, morphogenesis, embryo-genesis, leaf senesce, organogenesis, embryogenesis, and fruit maturation of the respective vegetable crop (Chen et al., 2019). Multiple abiotic stresses can be regulated by the application of varying concentrations of polyamines in horticultural crops, especially vegetable crops. Abiotic stresses can be regulated by the alteration of numerous processes of plants with a spray of polyamines available in the markets globally, as reported by Kamran et al. (2019). Moreover, the exogenous application of spermidine revealed good outcomes for tomato seedlings grown under stressful conditions. Moreover, the application of spermidine also enhanced the concentration of polyamine compounds within cells and compartments, especially in the root zone of tomato seedlings. The higher concentration of spermidine can be effective for tomato plants growing under saline conditions. The differentiation of ions and their translocation to other plant parts can be improved by supplementing polyamines (Hu et al., 2012). Exogenous application of spermidine is found to be effective for the improvement of plant growth, chlorophyll content, proline level, and different sugars, as reported by Zapata et al. (2004). Furthermore, it has also been reported that the reduction in ROS, MDA, and H_2_O_2_ was also measured in tomato plants. Pepper seeds were treated with different polyamines (spermine, putrescine, and spermidine), and it has been studied that the improved rate of germination, higher germination index, and early germination were recorded in treated seeds as compared to non-treated seeds. Similarly, in another study by Wu et al. (2018), it was revealed that the application of polyamines in cucumber seedlings improved crop performance under stressful conditions. Ormrod and Beckerson (1986) reported that polyamines are stress-relieving molecules as in the tomato for higher yield. The reduction and balance in the generation of oxidative stress markers, i.e., ROS, H_2_O_2_, MDA, free radicles, and movement of electrons, indicate the reduced stress in plants. Therefore, it has been studied that PA is an appropriate hormone for the improvement of endogenous hormones and also improved the activation of scavengers of toxic compounds.

### Ascorbic acid

This contributed to the regulation of biosynthesis of ascorbates within the plant body. It is involved in the detoxification and compartmentation of H_2_O_2_ and MDA activities. Ascorbic acid is an important phytohormone necessary for sustainable vegetable production globally, grown under drought and salt stress conditions. The increased concentration of ascorbic acid on lettuce revealed that ascorbic acid is also effective for increasing the fresh and dry weights of lettuce and the number of leaves, which are considered yield-contributing factors against salinity. Seed germination is disturbed due to stressful conditions. Therefore, the exogenous application of AsA significantly enhanced seed germination with the endogenous improvement of ascorbates, which further activates the scavengers of toxic bioactive molecules within the plant cells. Hence, the initiation of seed germination is regulated with supplemental AsA as studied by Akram et al. (2017). Moreover, its application had a good role in the balance and neutralization of free radicals and toxic ROS generated within the plant cells. The exogenous application of ascorbic acid effectively improves the endogenous ascorbic acid content.

### Abscisic acid

Its production is enhanced due to low moisture availability in the root zone of plants (Seiler et al., 2011; Lim et al., 2015). The enhanced production of abscisic acid (ABA) adversely affected plant growth and yield by producing nutritional imbalances (Sreenivasulu et al., 2012). The optimum production of ABA regulates the osmotic stress conditions (Ali et al., 2021a; Ali et al., 2021b). Induced levels of Na^+^ and ABA are the main causes of nutrient uptake restriction and nutrient translocation from roots toward leaves for food synthesis (Soma et al., 2021). Vegetables faced a reduction in stem and leaf, cell membrane injury, chlorophyll instability, lipid peroxidation, low water potential, degradation of photosynthetic pigments, poor gas exchange, higher Na^+^, Cl^−^, ABA, reduced K^+^, turgidity in leaf, osmolyte generation, and ROS scavenger production under stress (Malhi et al., 2021).

### Melatonin

Melatonin (MLE) is a stress-reducing molecule by exogenous supplementation. It is involved in the improvement of seed germination, the proliferation of roots, better flowering, fruit set and enlargement, fruit ripening, shelf life, and quality as studied by Wang et al. (2021). Drought and salinity stresses can be mitigated by the supplemental spray of MLE on many vegetable crops because of MLE’s multifaceted functions (Wang et al., 2013; Qi et al., 2018; Altaf et al., 2022b). MLE has the good capability to scavenge toxic ROS, MDA, H_2_O_2_, and electrolyte leakage as studied by Wang et al. (2012). The enhanced level of endogenous MLE has the capability to mitigate challenges that occur from drought and salinity in agricultural crops as reported by Nawaz et al. (2018). Restricted translocation of minerals (macronutrients and micronutrients) is upregulated by supplemental application of MLE. Moreover, the uptake and absorption of minerals by roots are regulated due to the application of appropriate melatonin levels. Oxidative and osmotic stresses are relieved by MLE due to the regulation of endogenous hormones and activation of scavengers of oxidative stress markers (Neha et al., 2021). The morphology of roots is improved regarding uptake, absorption, and further translocation toward other plant parts by supplemental MLE. Drought and salinity are involved in the disruption of plant metabolism. Therefore, disturbance in the plant metabolism is an indication of a stress situation. The activation of oxidative stress markers like ROS, H_2_O_2_, and MDA is reduced, and their scavengers (enzymatic, non-enzymatic, and osmolytes) are activated. Therefore, the availability of nutrients to plants is imperative especially when growing under stressful conditions. Hence, MLE can be a suitable method for the alleviation of drought and salinity in vegetable crops.

**Figure 3:**
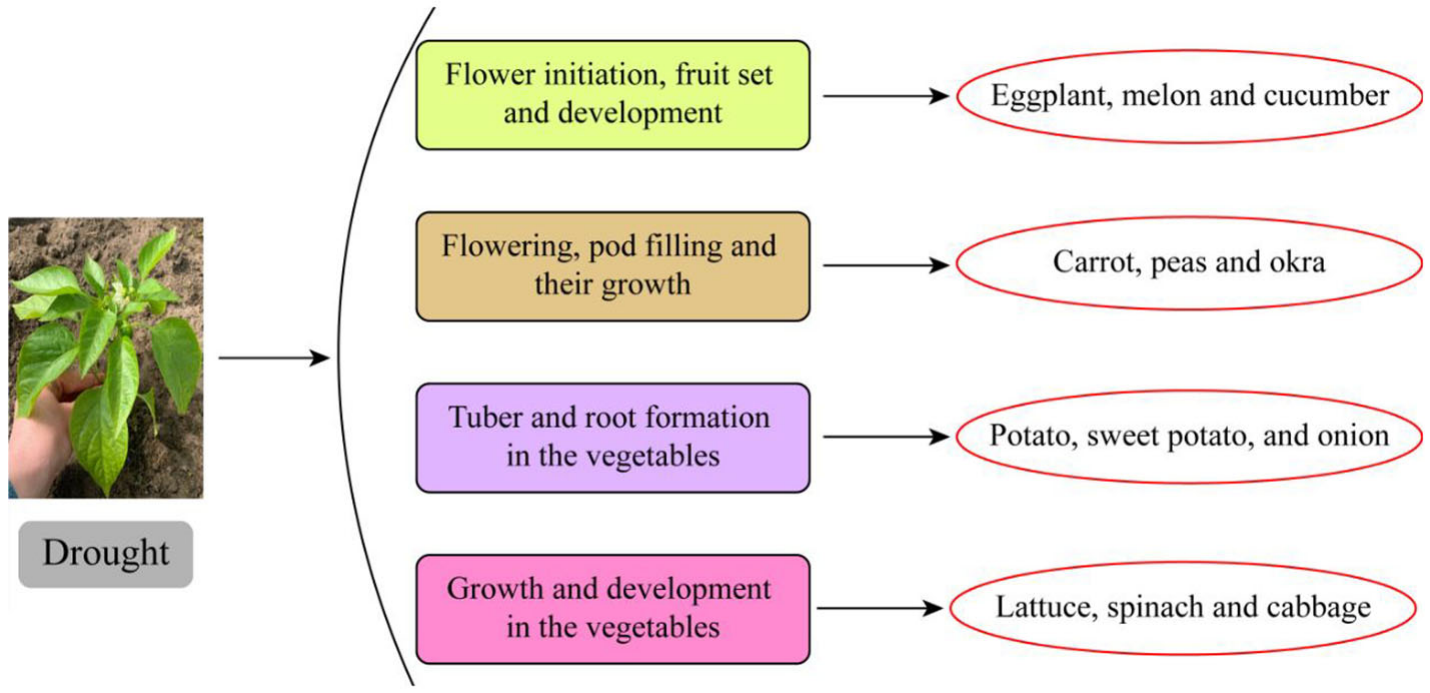
Impact of phytohormones on different vegetable crops growing under drought and salinity stresses.

Hormonal regulations are necessary to enhance vegetable crop tolerance against drought and salinity stress conditions (**Figure 3**). Different concentrations of antioxidant sprays improved crop performance by modulation of physiological and biochemical mechanisms in sweet potatoes (Lin et al., 2006) (**Table 2**).

**Table 2:** The role of exogenous phytohormones against drought and salinity stresses in vegetable production.

Stress type	Phytohormones	Vegetable crop	Key findings	References
Salinity	Salicylic acid	Pea	0.4 mM of salicylic acid enhances growth by improving the defense system	Jangid and Dwivedi (2016)
Salinity	Ascorbic acid	Lettuce	0, 100, 200, 300, and 400 mg/L improved lettuce performance with improved yield	Jangid and Dwivedi (2016)
Drought	Osmoprotectants	Tomato	50%–57% of field capacity also increased the level of salts in plants. Germination was very poor in seeds	Jangid and Dwivedi (2016)
Drought	Salicylic acid	Tomato	10–5 M improved seedling growth under 10 days of water-holding capacity	Hayat et al. (2008)
Drought	Melatonin	Cucumber	100 μM significantly improved growth, yield, and defense mechanism	Zhang et al. (2013)
Salinity	Polyamines (spermidine)	Cucumber	Adverse effects due to 50 mM of NaCl can be regulated by the application of polyamines such as spermidine	Duan et al. (2008)
Drought	Methyl jasmonate	Sweet potatoes	13 µM/L of jasmonates improved growth, yield, and quality characters	Yoshida et al. (2020)
Drought	Jasmonic acid	Potato	Overexpression of StJAZ1 resulted in decreased relative leaf water potential in the plants MDA and lipid peroxidation were enhanced. Jasmonic acid is effective to reduce the production of MDA and lipid peroxidation	Jing et al. (2022)
Drought	Mannitol and methyl jasmonate	Pepper	Regulates signaling and antioxidant defense potential in plants	Ma et al. (2021)

MDA, malondialdehyde.

## Conclusion and prospects

In the present study, it has been explored that modulation of physiological and biochemical mechanisms is necessary for sustainable production of vegetable crops growing under drought and salinity stresses. Exogenous application of phytohormones is necessary for the improvement of vegetable growth, yield, photosynthetic pigments, minerals nutrient content, and defense-related characteristics. It has been concluded that phytohormones are necessary for the sustainable production of vegetable crops.

* Climate change, urbanization, and industrial zones are depleting and polluting water resources. Water shortage is going to worsen. To feed a huge population, it is necessary to develop management approaches to obtain higher vegetable production with limited water resources.
* Elevated drought and salinity conditions severely affect the productivity of vegetable crops. In this situation, phytohormones are considered a supportive strategy for the sustainable production of vegetable crops in the current scenario.
* To achieve zero hunger, it is necessary to elevate drought and salinity tolerance in vegetables. Moreover, the development of tolerant landraces is also a present need.
* Exploration of molecular basis, i.e., genome characterization, QTL mapping, marker-assisted selection (MAS), genome editing, genetic transformation, and genome sequencing are also imperative for the development of tolerant germplasm of vegetable crops.

## Author contributions

JC: conceptualization, literature survey, writing major original draft, and review structure. XP: literature survey, writing—review and editing, and figure designing. All authors contributed to the article and approved the submitted version.
